# Supplementary material for: A model for network-based identification and pharmacological targeting of aberrant, replication-permissive transcriptional programs induced by viral infection
Source: Commun Biol. 2022 Jul 19;5:714. doi: 10.1038/s42003-022-03663-8 (PMC9296638; doi:10.1038/s42003-022-03663-8)
Supplement: Supplementary file 2 — Supplementary Information [file 42003_2022_3663_MOESM2_ESM.pdf]

Supplementary Information for

A model for network-based identification and pharmacological targeting of aberrant, replication-permissive transcriptional programs induced by viral infection

Pasquale Laise, Megan L. Stanifer, Gideon Bosker, Xiaoyun Sun, Sergio Triana, Patricio Doldan, Federico La Manna, Marta De Menna, Ronald B. Realubit, Sergey Pampou, Charles Karan, Theodore Alexandrov, Marianna Kruithof-de Julio, Andrea Califano, Steeve Boulant and Mariano J. Alvarez

To whom correspondence should be addressed:

Andrea Califano: ac2248@cumc.columbia.edu

Steeve Boulant: s.boulant@ufl.edu

Mariano Alvarez: malvarez@darwinhealth.com

This PDF file includes:

Supplementary Figures 1 to 9

Supplementary Tables 1 to 3

Supplementary References

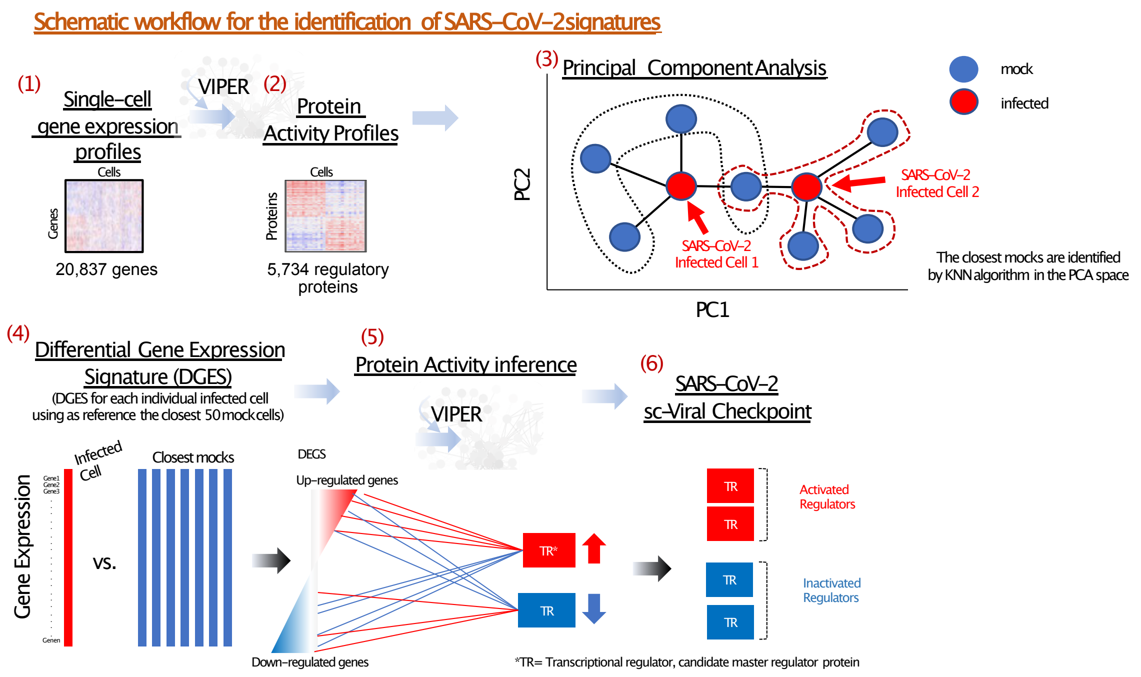

Supplementary Figure 1. Diagram showing the workflow used to compute the protein activity signatures induced by SARS-CoV-2 infection from scRNA-Seq data. Related to Figure 2 and methods. (1) Normalized single-cell gene expression profiles for all cells of the same model (i.e. Calu3, H1299, colon and ileum) were transformed to differential gene expression signatures by applying the z-score procedure (subtracting their mean and dividing by their standard deviation). (2) Protein activity signatures were then computed with the VIPER algorithm using lineage-matched context-specific regulatory networks. (3) A principal component analysis (PCA) was performed on these VIPER-inferred protein activity profiles. (4) For each infected cell the closest 50 mock cells in the PCA space were selected as reference to compute a SARS-CoV-2 induced differential gene expression signature. (5) The VIPER algorithm was then applied to these SARS-CoV-2 induced differential gene expression signatures to infer SARS-CoV-2 induced protein activity signatures. (6) The protein activity signatures were averaged and the most differentially activated and inactivated proteins were considered as candidate MRs.

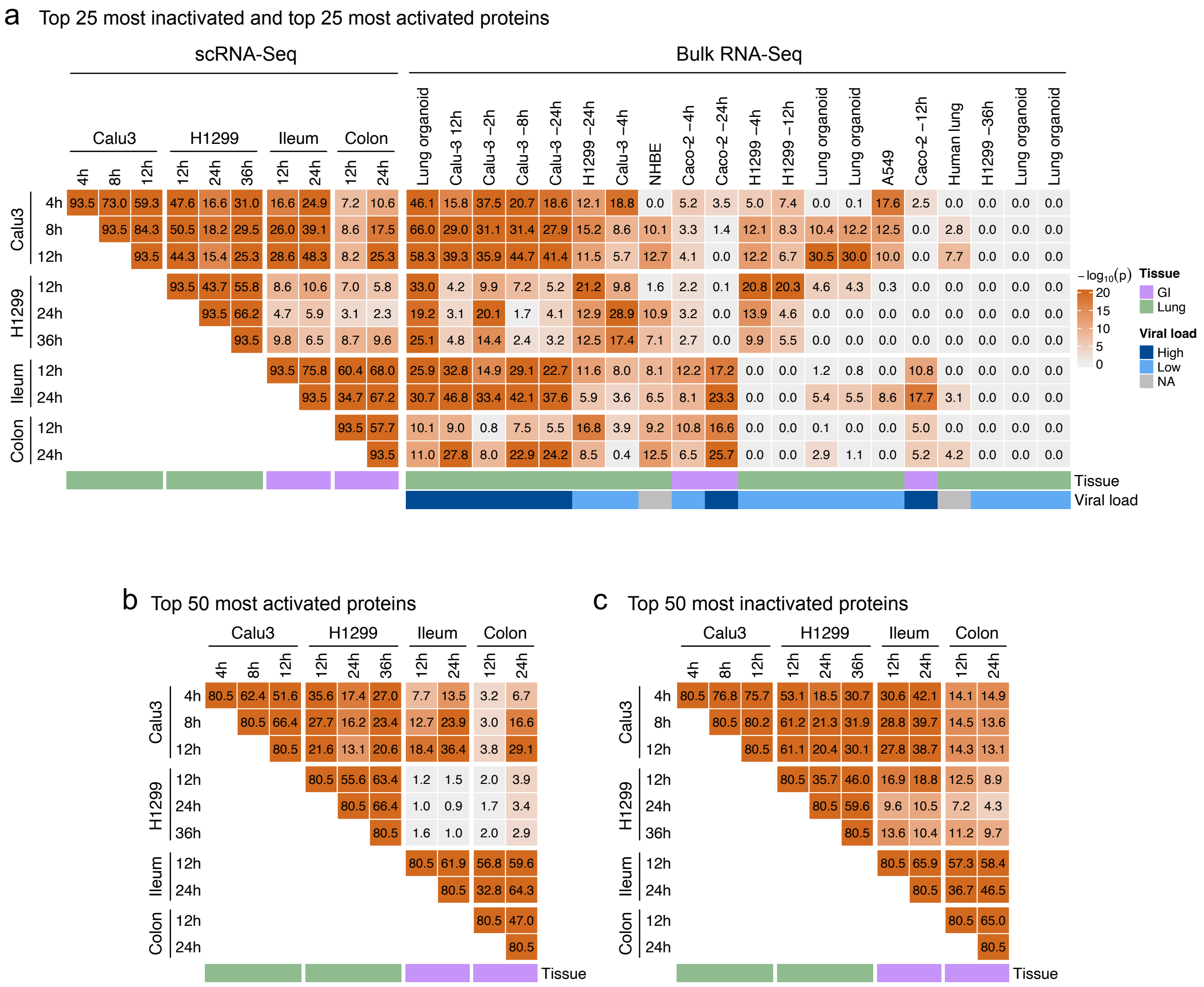

Supplementary Figure 2. Conservation of VIPER-inferred Viral Checkpoint. Related to Figure 2. a. Heatmap showing the conservation across single-cell and bulk-tissue samples. Results are expressed as -log$_{10}$(p-value), estimated by the reciprocal enrichment of the 25 most activated and 25 most inactivated proteins in each signature using the aREA algorithm as implemented in the viperSimilarity function of the VIPER package. **b-c.** Conservation specifically for the top 50 most activated proteins (b) and most inactivated proteins (c) in response to SARS-CoV-2 infection between time points and models profiled at the single-cell level.

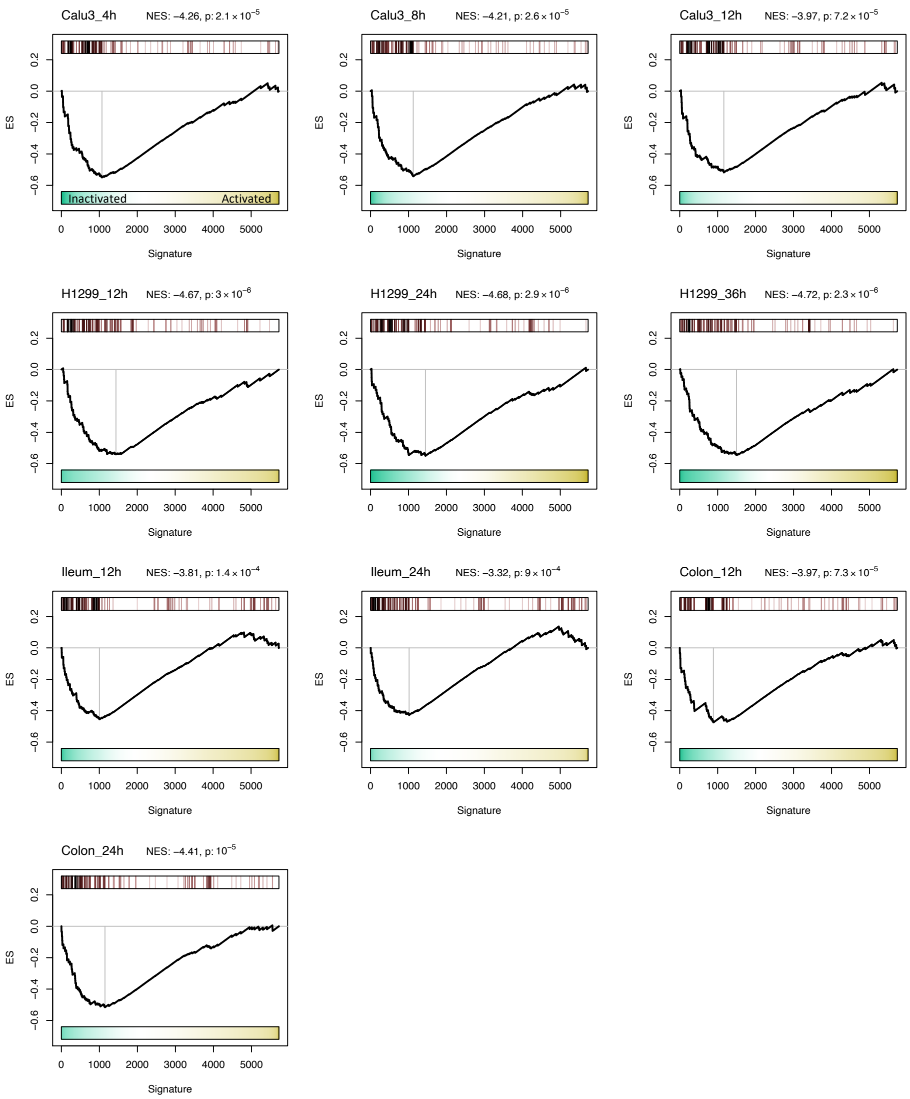

Supplementary Figure 3. Enrichment of host factors known to physically interact with SARS-CoV-2 proteins on the host proteins differentially active in response to viral infection. Related to Figure 2. GSEA showing the enrichment for the SARS-CoV-2 interacting proteins in the individual SARS-CoV-2 induced protein activity signatures. NES and p-values were estimated by two-tailed test and 1,000 permutations.

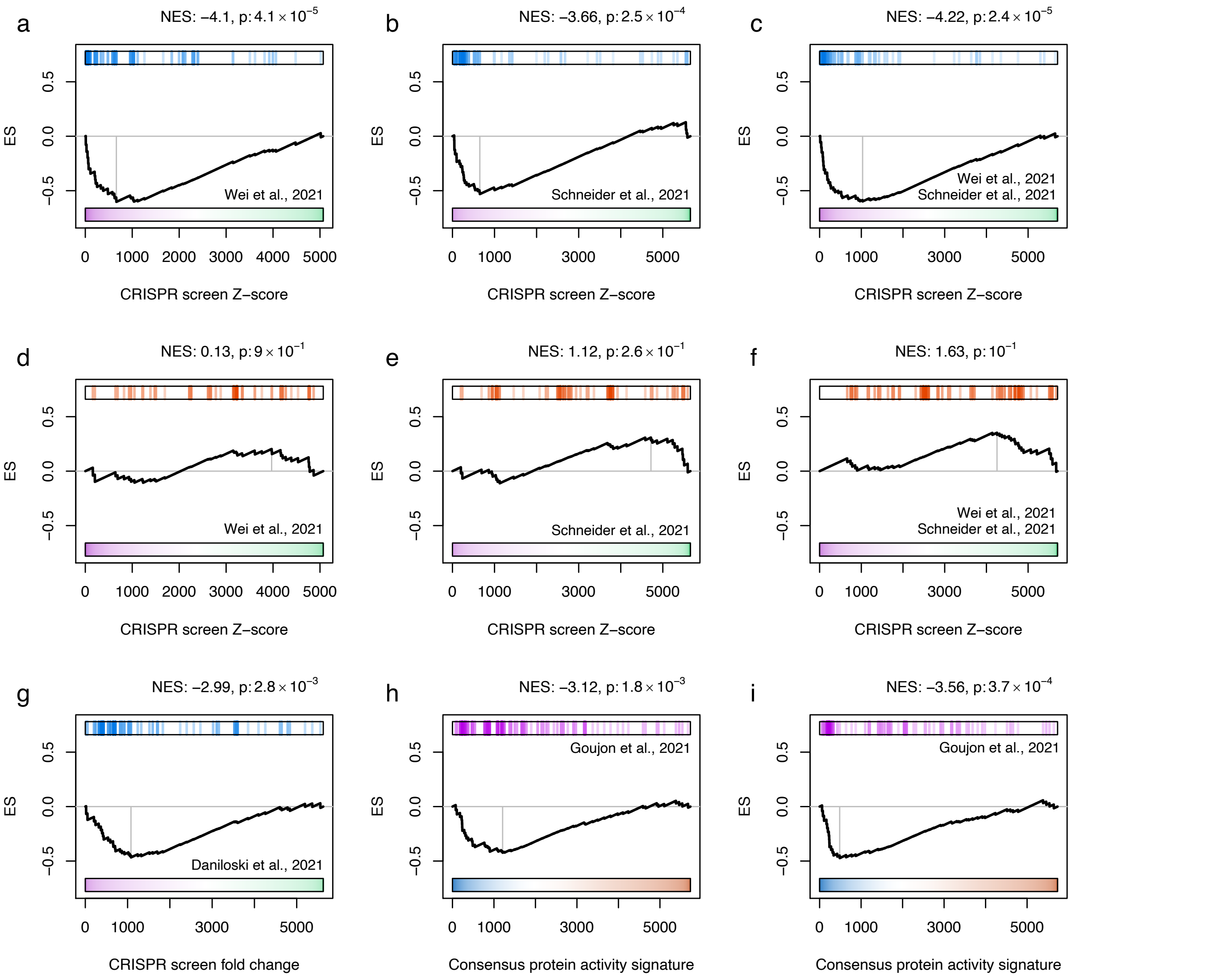

Supplementary Figure 4. Enrichment of candidate SARS-CoV-2 infection MR proteins on host factors essential for SARS-CoV-2 infectivity. Related to Figure 2. Host proteins inactivated by SARS-CoV-2 infection are enriched in antiviral essential factors. (a-f) GSEA showing the enrichment of the top 50 most inactivated proteins in response to SARS-CoV-2 infection (inactivated candidate MR proteins) on the antiviral essential genes identified by CRISPR screens in Vero cells [1] (a), Huh-7.5 cell [2] (b), and their integration (Stouffer's integration of z-scores, c), but no enrichment of the top 50 most activated proteins in response to SARS-CoV-2 infection (activated candidate MR proteins) on the pro-viral essential genes identified by the same studies (d-e), or their integration (f). (g) GSEA showing enrichment of the 50 most inactivated candidate MRs on antiviral essential genes identified by CRISPR screen in A549-

ACE2 cells [3]. (h-i) GSEA showing enrichment of antiviral essential genes identified by CRISPR screens in Calu-3 (h) and Caco-2 cells [4] (i) among the consensus most inactivated proteins in response to SARS-CoV-2 infection of lung adenocarcinoma cell lines and GI-organoids.

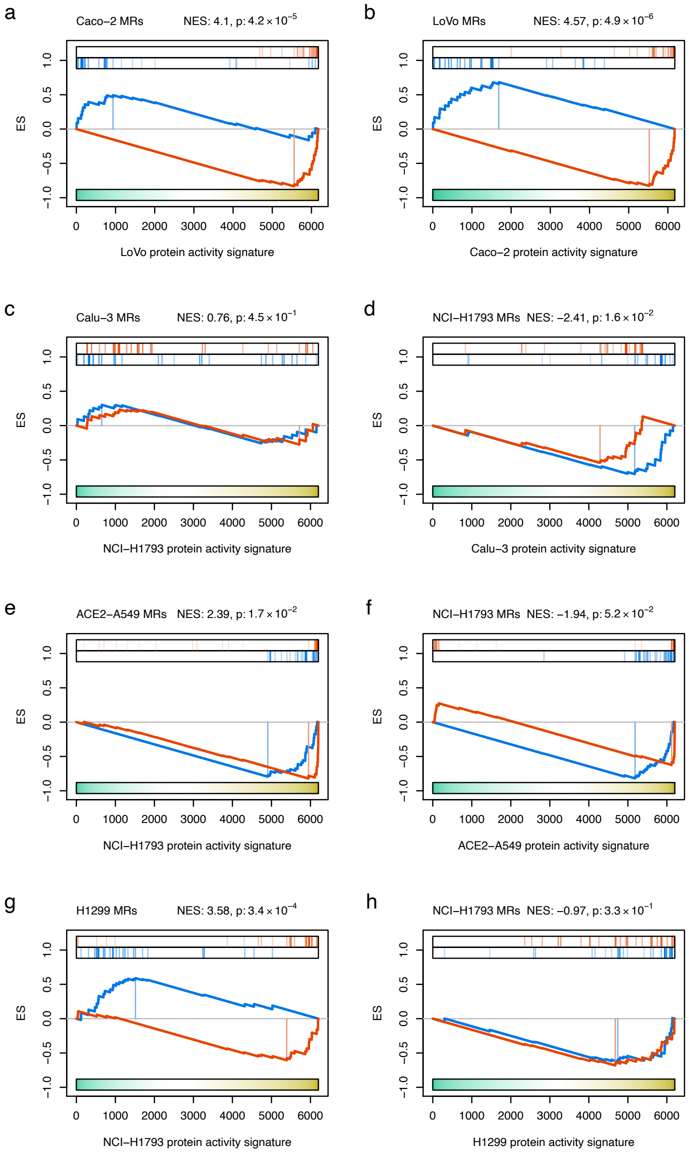

Supplementary Figure 5. Conserved activity of MR proteins between cell line models susceptible to SARS-CoV-2 infection (Caco-2, Calu-3, ACE2-A549 and H1299) and the lineage context-matched cell lines included in the drug perturbation PANACEA resource (LoVo and NCI-H1793). Related to Figure 2-3. a. GSEA for the enrichment of the Caco-2 top 25 most activated and top 25 most inactivated proteins in the LoVo protein activity signature. **b.** GSEA for the enrichment of the LoVo top 25 most activated and top 25 most inactivated proteins in the Caco-2 protein activity signature. **c.** GSEA for the enrichment of the Calu-3 top 25 most activated and top 25 most inactivated proteins in the NCI-H1793 protein activity signature. **d.** GSEA for the enrichment of the NCI-H1793 top 25 most activated and top 25 most inactivated proteins in the Calu-3 protein activity signature. **e.** GSEA for the enrichment of the ACE2-A549

top 25 most activated and top 25 most inactivated proteins in the NCI-H1793 protein activity signature. **f.** GSEA for the enrichment of the NCI-H1793 top 25 most activated and top 25 most inactivated proteins in the ACE2-A549protein activity signature. **g.** GSEA for the enrichment of the H1299 top 25 most activated and top 25 most inactivated proteins in the NCI-H1793 protein activity signature. **h.** GSEA for the enrichment of the NCI-H1793 top 25 most activated and top 25 most inactivated proteins in the H1299 protein activity signature. Normalized enrichment score (NES) and p-value were estimated by two-tailed test and 1,000 permutations.

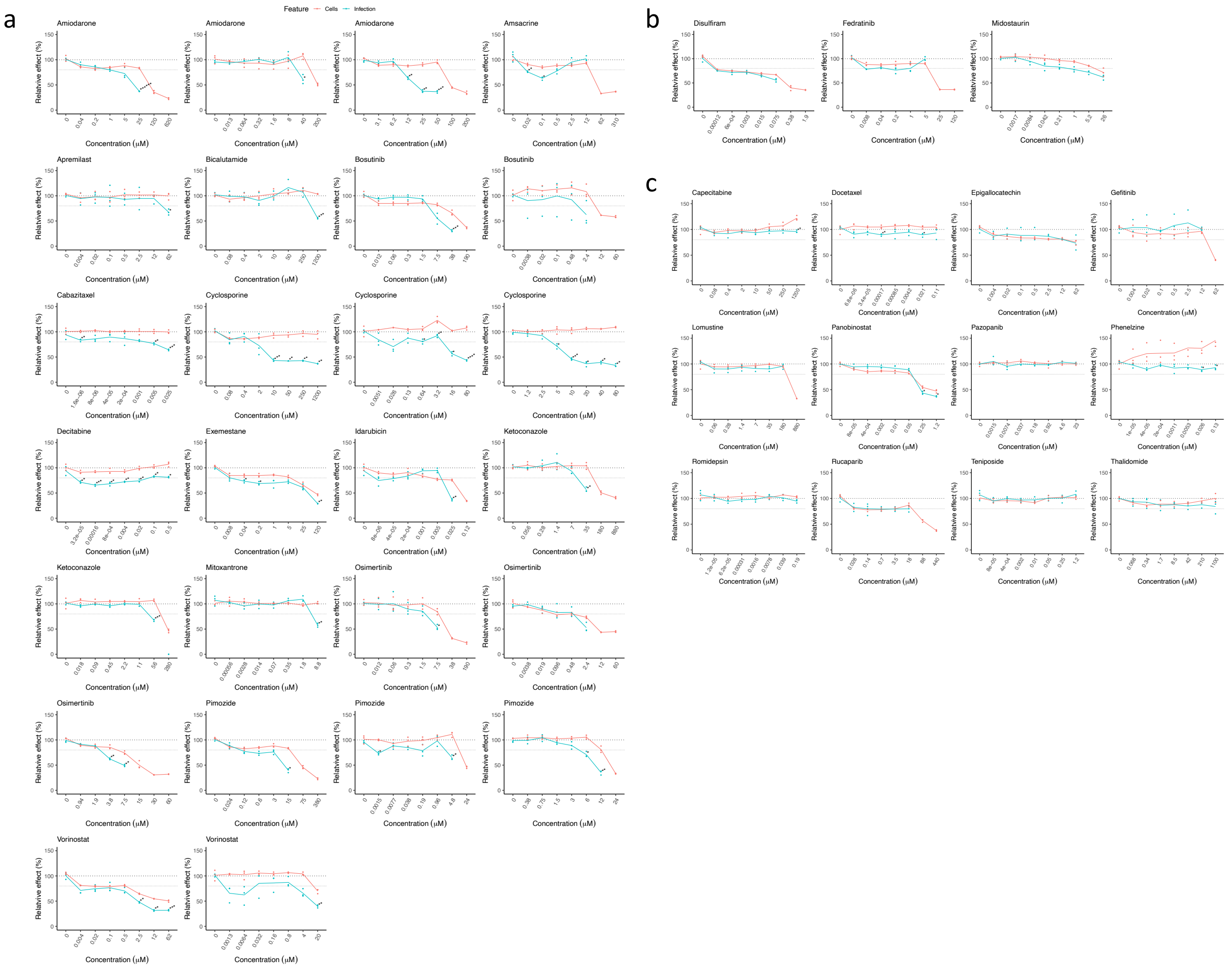

Supplementary Figure 6. Experimental evaluation of the antiviral effect of FDA-approved drugs in Caco-2 cells. Related to Figure 4 and Supplementary Table 3. a. 15 of the 18 drugs predicted by ViroTreat showing significant antiviral effect (FDR < 0.05 and ≥ 20% viral replication decrease). **b.** 3 of the 18 drugs predicted by ViroTreat showing no significant antiviral effect. **c.** 12 drugs not significant by ViroTreat (p ≥ 0.01) selected as putative negative controls. The scatter-plots show the effect of each drug—SARS-CoV-2 replication shown in cyan and cell viability in red—relative to vehicle control (y-axis), assayed at different concentrations (x-axis) in triplicate. The lines indicate the average across replicates. * $p < 0.05$, ** $p < 0.01$, *** $p < 0.001$, **** $p < 10^{-4}$, ****** $p < 10^{-6}$, 1-tailed Student's t-test, BC.

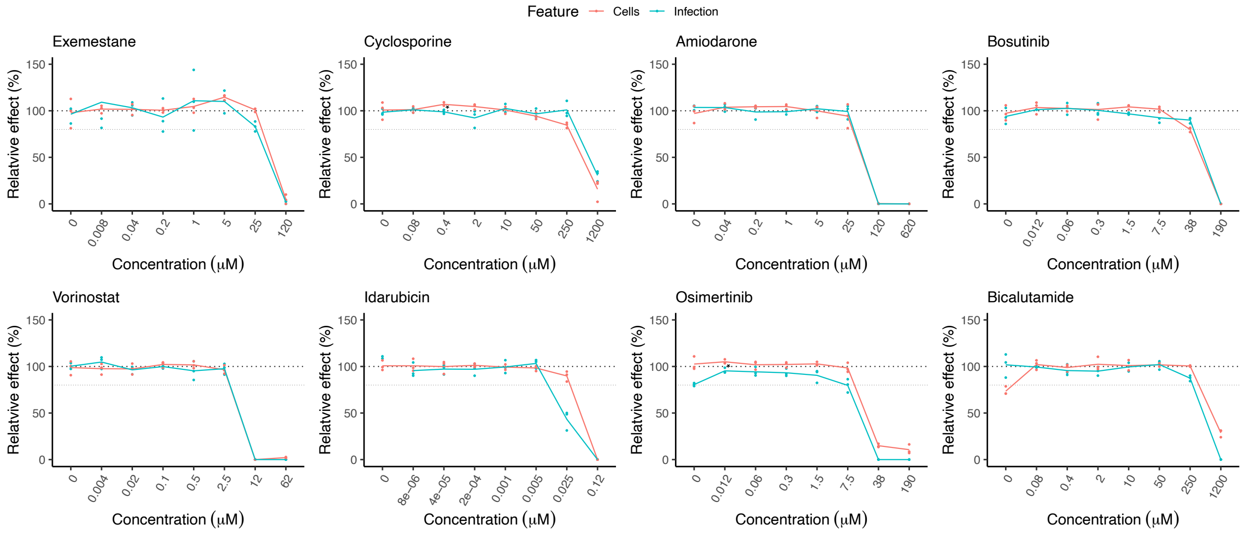

Supplementary Figure 7. Experimental evaluation of 8 drugs, predicted by ViroTreat and showing the strongest SARS-CoV-2 antiviral effect in Caco-2 cells, for their effect on rotavirus replication. Related to Figure 4 and Supplementary Table 3. The scatter-plots show the effect of each drug—rotavirus replication shown in cyan and cell viability in red—relative to vehicle control (y-axis), assayed at different concentrations (x-axis) in triplicate. The lines indicate the average across replicates. * $p < 0.05$, 1-tailed Student's t-test, BC.

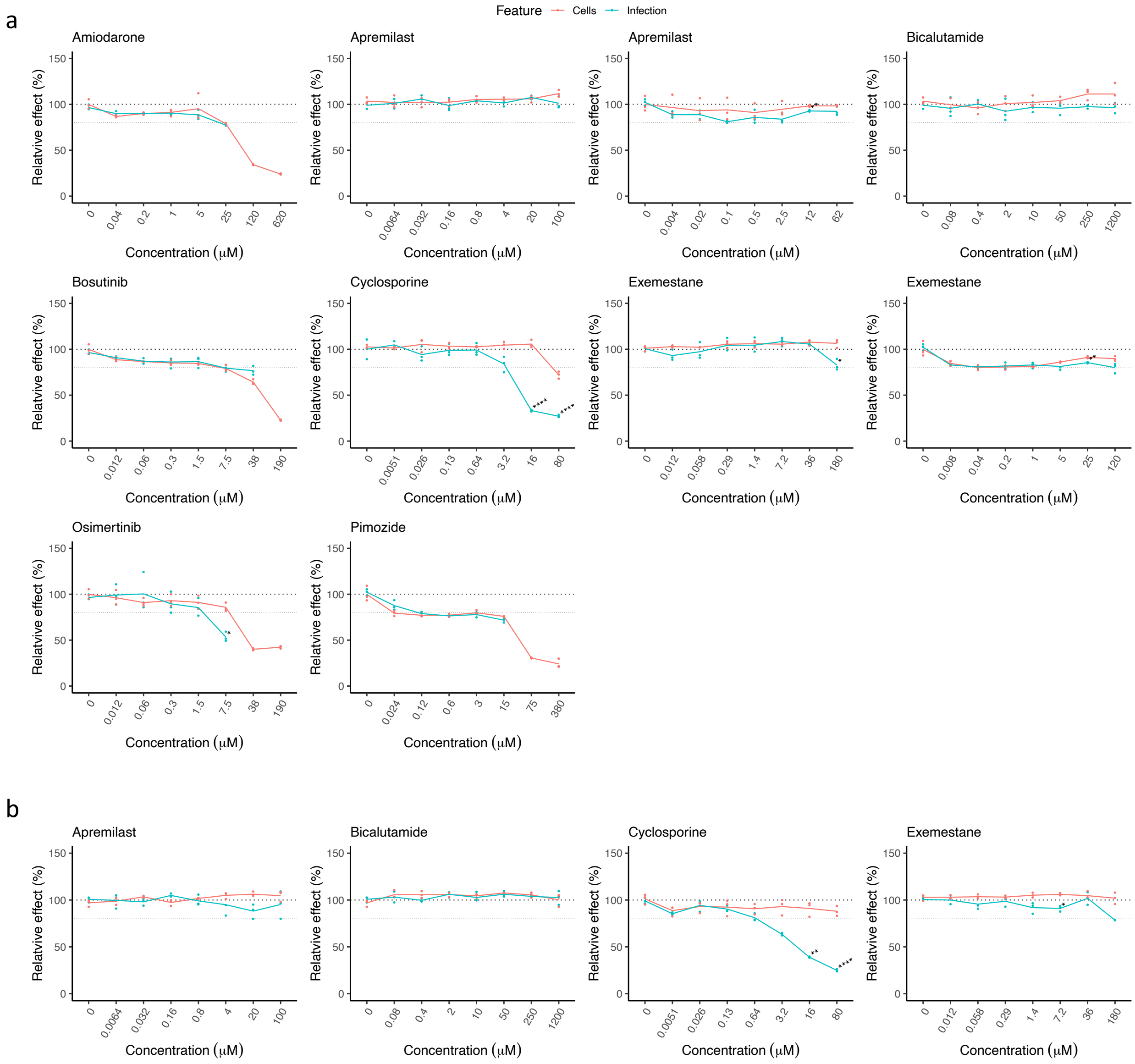

Supplementary Figure 8. Experimental evaluation of the antiviral effect of FDA-approved drugs in lung adenocarcinoma cell lines. Related to Figure 4 and Supplementary Table 3. A set of drugs, predicted by ViroTreat for the GI context and with validated antiviral effect in Caco-2 cells were evaluated in Calu-3 (a) and A549-ACE2 (b) cells. The scatter-plots show the effect of each drug—SARS-CoV-2 replication shown in cyan and cell viability in red—relative to vehicle control (y-axis), assayed at different concentrations (x-axis) in triplicate. The lines indicate the average across replicates. * $p < 0.05$, ** $p < 0.01$, **** $p < 10^{-4}$, 1-tailed Student's t-test, BC.

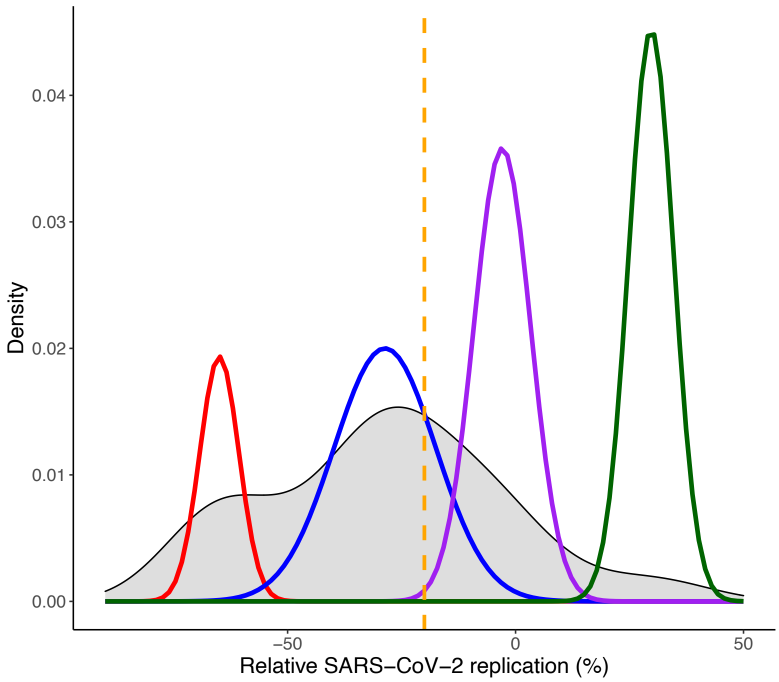

Supplementary Figure 9. Distribution for the relative effect of the evaluated drugs on SARS-CoV-2 replication. Related to Figure 4. Distribution density for the relative effect of the drugs, expressed as percentage, on SARCS-CoV-2 replication in Caco-2 cells (grey) and a mixture of 4 Gaussian models (colored curves) fitted to it. The dashed orange vertical line represents the threshold of 20% used as additional criteria when considering the antiviral effect of a drug.

Supplementary Table 1: SARS-CoV-2 host cell RNA-Seq and scRNA-Seq datasets.

Model	*Type of Data*	*Publication*	*Source*
Calu3	Bulk RNASeq	Wyler et al.[1]	(GEO) GSE148729
H1299	Bulk RNASeq	Wyler et al.[1]	(GEO) GSE148729
Caco2	Bulk RNASeq	Wyler et al.[1]	(GEO) GSE148729
A549	Bulk RNASeq	Blanco Melo et al.[2]	(GEO) GSE147507
Lung Organoids	Bulk RNASeq		(GEO) GSE160435
NHBE	Bulk RNASeq	Blanco Melo et al.[2]	(GEO) GSE147507
Human lung	Bulk RNASeq	Blanco Melo et al.[2]	(GEO) GSE147507
Calu3	scRNASeq	Wyler et al.[1]	(GEO) GSE148729
H1299	scRNASeq	Wyler et al.[1]	(GEO) GSE148729
Ileum	scRNASeq	Triana et al.[3]	Boulant Lab
Colon	scRNASeq	Triana et al.[3]	Boulant Lab
Vero6	CRISPRcas9	Wei et al.[4]	Supplementary Data
A549	CRISPRcas9	Daniloski et al.[5]	Supplementary Data
Huh-7.5	CRISPRcas9	Wang et al.[6]	Supplementary Data
Huh-7.5	CRISPRcas9	Schneider et al.[7]	Supplementary Data

[1]Wyler, E., et al. (2021). iScience **24**(3): 102151.
[2]Blanco-Melo, D., et al. (2020). Cell **181**(5): 1036-1045 e1039.
[3]Triana, S., et al. (2021). Mol Syst Biol **17**(4): e10232.
[4]Wei, J., et al. (2021). Cell **184**(1): 76-91 e13.
[5]Daniloski, Z., et al. (2021). Cell **184**(1): 92-105 e116.
[6]Wang, R., et al. (2021). Cell **184**(1): 106-119 e114.
[7]Schneider, W. M., et al. (2021). Cell **184**(1): 120-132 e114.

Supplementary Table 2: Organoids' culture media.

Compound	*Final concentration*
**Basal media**	
Ad DMEM/F12	
+GlutaMAX	
+HEPES	
+P/S	
L-WRN	50% by volume
B27	1:50
N-acetyl-cysteine	1 mM
EGF	50 ng/mL
A83-01	500 nM
IGF-1	100 ng/mL
FGF basic	50 ng/mL
Gastrin	10 mM
**Differentiation Media**	
Ad DMEM/F12	
+GlutaMAX	
+HEPES	
+P/S	
B27	1:50
N-acetyl-cysteine	1 mM
R-spondin	5% by volume
Noggin	50 ng/mL
EGF	50 ng/mL
Gastrin	10 mM
A83-01	500 nM

Supplementary Table 3: PCR primers.

Gene name	*Species*	*Forward sequence*	*Reverse sequence*
HPRT1	Human	cct ggc gtc gtg att agt gat	aga cgt tca gtc ctg tcc ata a
COV1	SARS-CoV-2	gcc tct tct gtt cct cat cac	aga cag cat cac cgc cat tg

Supplementary References

1 Wei, J. *et al.* Genome-wide CRISPR Screens Reveal Host Factors Critical for SARS-CoV-2 Infection. *Cell* **184**, 76-91 e13, doi:10.1016/j.cell.2020.10.028 (2021).

2 Schneider, W. M. *et al.* Genome-Scale Identification of SARS-CoV-2 and Pan-coronavirus Host Factor Networks. *Cell* **184**, 120-132 e114, doi:10.1016/j.cell.2020.12.006 (2021).

3 Daniloski, Z. *et al.* Identification of Required Host Factors for SARS-CoV-2 Infection in Human Cells. *Cell* **184**, 92-105 e116, doi:10.1016/j.cell.2020.10.030 (2021).

4 Goujon, C. *et al.* Bidirectional genome-wide CRISPR screens reveal host factors regulating SARS-CoV-2, MERS-CoV and seasonal HCoVs. *Res Sq*, doi:10.21203/rs.3.rs-555275/v1 (2021).
