# Supplementary material for: A model for network-based identification and pharmacological targeting of aberrant, replication-permissive transcriptional programs induced by viral infection
Source: Commun Biol. 2022 Jul 19;5:714. doi: 10.1038/s42003-022-03663-8 (PMC9296638; doi:10.1038/s42003-022-03663-8)
Supplement: Supplementary file 3 — Description of Additional Supplementary Files [file 42003_2022_3663_MOESM3_ESM.pdf]

Description of Additional Supplementary Files

File name: Supplementary Data 1
Description: Protein activity signatures induced by SARS-CoV-2 infection. Values correspond to Normalized Enriched Score (NES) computed by VIPER.

File name: Supplementary Data 2
Description: Drugs library, ViroTreat and focused validation screen results.

File name: Supplementary Data 3
Description: R source code for ViroTreat (see supplementary data file viroTreat.r).

File name: Supplementary Data 4
Description: Source data corresponding to Figs. 4b and c.

File name: Supplementary Data 5
Description: Source data corresponding to Fig. 4d.
