# Supplementary material for: A model for network-based identification and pharmacological targeting of aberrant, replication-permissive transcriptional programs induced by viral infection
Source: Commun Biol. 2022 Jul 19;5:714. doi: 10.1038/s42003-022-03663-8 (PMC9296638; doi:10.1038/s42003-022-03663-8)
Supplement: Supplementary file 9 — Reporting Summary [file 42003_2022_3663_MOESM9_ESM.pdf]

nature research

Corresponding author(s): Andrea Califano, Steeve Boulant, Mariano J. Alvarez

Last updated by author(s): Jun 23, 2022

Reporting Summary

Nature Research wishes to improve the reproducibility of the work that we publish. This form provides structure for consistency and transparency in reporting. For further information on Nature Research policies, see our Editorial Policies and the Editorial Policy Checklist.

Statistics

For all statistical analyses, confirm that the following items are present in the figure legend, table legend, main text, or Methods section.

n/a	Confirmed	
☐	☒	The exact sample size (n) for each experimental group/condition, given as a discrete number and unit of measurement
☐	☒	A statement on whether measurements were taken from distinct samples or whether the same sample was measured repeatedly
☐	☒	The statistical test(s) used AND whether they are one- or two-sided<br>*Only common tests should be described solely by name; describe more complex techniques in the Methods section.*
☐	☒	A description of all covariates tested
☐	☒	A description of any assumptions or corrections, such as tests of normality and adjustment for multiple comparisons
☐	☒	A full description of the statistical parameters including central tendency (e.g. means) or other basic estimates (e.g. regression coefficient) AND variation (e.g. standard deviation) or associated estimates of uncertainty (e.g. confidence intervals)
☐	☒	For null hypothesis testing, the test statistic (e.g. F, t, r) with confidence intervals, effect sizes, degrees of freedom and P value noted<br>*Give P values as exact values whenever suitable.*
☐	☒	For Bayesian analysis, information on the choice of priors and Markov chain Monte Carlo settings
☒	☐	For hierarchical and complex designs, identification of the appropriate level for tests and full reporting of outcomes
☒	☐	Estimates of effect sizes (e.g. Cohen's d, Pearson's r), indicating how they were calculated

Our web collection on statistics for biologists contains articles on many of the points above.

Software and code

Policy information about availability of computer code

Data collection	CellProfiler v4.2.0 (cellprofiler.org)
Data analysis	R v4.0.5 (r-project.org) and R packages: viper v1.24.0; ComplexHeatmap v2.6.2; graph v1.68.0; ggplot2 v3.3.3; pROC v1.17.0.1; mixtools v1.2.0MASS v7.3-54; kernlab v0.9-29; e1071 v1.7-6; KernSmooth v2.8; aracne.networks v1.20.0

For manuscripts utilizing custom algorithms or software that are central to the research but not yet described in published literature, software must be made available to editors and reviewers. We strongly encourage code deposition in a community repository (e.g. GitHub). See the Nature Research guidelines for submitting code & software for further information.

Data

Policy information about availability of data

All manuscripts must include a data availability statement. This statement should provide the following information, where applicable:
- Accession codes, unique identifiers, or web links for publicly available datasets
- A list of figures that have associated raw data
- A description of any restrictions on data availability

Availability of SARS-CoV-2 host cell RNA-Seq and scRNA-Seq datasets is indicated in Supplementary Table 1. The drug perturbational dataset (PLATE-seq) for the colorectal adenocarcinoma (LoVo) model is available from Douglass et.al. (PMID 35106508). The context-specific interactomes are available from Bioconductor as part of the arcane.networks package for R (https://www.bioconductor.org). The source data for the plots are available as Supplementary Data 4 and 5.

Field-specific reporting

Please select the one below that is the best fit for your research. If you are not sure, read the appropriate sections before making your selection.

☒ Life sciences ☐ Behavioural & social sciences ☐ Ecological, evolutionary & environmental sciences

For a reference copy of the document with all sections, see nature.com/documents/nr-reporting-summary-flat.pdf

Life sciences study design

All studies must disclose on these points even when the disclosure is negative.

Sample size	At least 3 independent replicates were performed per drug treatment arm. No power analysis was used.
Data exclusions	No data was excluded from the analysis
Replication	Reproducibility of SARS-CoV-2-induced regulatory protein activity signatures was evaluated across different time points, cell lines and organoid models. Drug effect on virus replication was evaluated using at least 3 independent replicates.
Randomization	Not relevant, cell line models and organoids were used for the experiments and controls were ran on the same multi-well plates.
Blinding	Not relevant. Data acquisition was automatically obtained by the same investigator performing the experiment.

Reporting for specific materials, systems and methods

We require information from authors about some types of materials, experimental systems and methods used in many studies. Here, indicate whether each material, system or method listed is relevant to your study. If you are not sure if a list item applies to your research, read the appropriate section before selecting a response.

Materials & experimental systems

n/a	Involved in the study	
☐	☒	Antibodies
☐	☒	Eukaryotic cell lines
☒	☐	Palaeontology and archaeology
☒	☐	Animals and other organisms
☒	☐	Human research participants
☒	☐	Clinical data
☒	☐	Dual use research of concern

Methods

n/a	Involved in the study	
☒	☐	ChIP-seq
☒	☐	Flow cytometry
☒	☐	MRI-based neuroimaging

Antibodies

Antibodies used	dsRNA (J2) produced from Scions (Distrubuted by Jena: https://www.jenabioscience.com/rna-technologies/rna-analysis-detection/dsrna-detection/rnt-sci-10010-anti-dsrna-monoclonal-antibody-j2)
Validation	This antibody has been validated to recognize dsRNA in cells caused by virus infection. The antibody has been used by several groups and has been validated as recognizing SARS-CoV-2 infected cells. See Stanifer, et al Cell Reports, 2020 for validation, FigureS1A (https://ars.els-cdn.com/content/image/1-s2.0-S2211124720308445-mmc1.pdf)

Eukaryotic cell lines

Policy information about cell lines

Cell line source(s)	Vero E6 (ATCC CRL-1586) and Caco-2 (ATCC HTB-37)
Authentication	Cells were purchased from official source (ATCC). Additionally Caco-2 cells were verified by https://www.multiplexion.de.
Mycoplasma contamination	All cell lines were confirmed negative for mycoplasma and were checked regularly using the MycoAlert mycoplasma detection kit from Lonza.
Commonly misidentified lines (See ICLAC register)	N/A
